# Effect of Lower Limb Venous Dilation on the Autonomic Cardiac Response among Healthy Young Men

**DOI:** 10.3390/healthcare11040548

**Published:** 2023-02-13

**Authors:** Daisuke Fujita, Yusuke Kubo, Tatsuya Tagawa

**Affiliations:** 1 Department of Physical Therapy, Faculty of Medical Science, Fukuoka International University of Health and Welfare, 3-6-40 Momochihama, Sawara-ku, Fukuoka-City 814-0001, Fukuoka, Japan; 2 Department of Rehabilitation, Kobori Orthopedic Clinic, 548-2 Nearaichou, Kita-ku, Hamamatsu-City 433-8108, Shizuoka, Japan

**Keywords:** heart rate variability, near-infrared spectroscopy, venous distention

## Abstract

Venous occlusion of the lower limbs, which simulates edema, can alter heart rate variability (HRV) by increasing feedback information from group III/IV sensory fibers. Our aim was to quantify this effect among healthy young men. The study group included 13 men (mean age, 20.4 years). Venous occlusion of the lower limbs was induced using a pressure cuff around both thighs. The effect of occlusion on autonomic cardiac response was quantified under occlusion pressures of 20, 60, and 100 mmHg. Compression was applied for 5 min. HRV was evaluated from changes in the low-frequency (LF) and high-frequency (HF) power of the electrocardiogram and the resulting LF/HF balance. Near-infrared spectroscopy of the leg was used to quantify the effects of occlusion on deoxyhemoglobin, measured as the area under the curve (HHb-AUC). The occlusion pressure of 100 mmHg induced a significant increase in the LF/HF ratio, compared to the baseline (*p* < 0.05). HHb-AUC was highest for the 100 mmHg occlusion pressure compared with the 20 and 60 mmHg pressures (*p* < 0.01). These findings indicate that venous dilation may elicit a shift towards sympathetic dominance in the autonomic balance.

## 1. Introduction

The afferent information of the skeletal muscles plays a key role in physiological responses during exercise [1,2]. These afferents are divided into two groups [2,3,4]: group III fibers that sense skeletal muscle stretch and pressure, and group IV fibers that sense the by-products of skeletal myocyte metabolism, leading to sympathetic activation and parasympathetic withdrawal. Feedback from group III/IV fibers increases cardiac output and blood pressure through the autonomic nervous system (ANS) to support effective oxygenation of the muscles [5,6]. Augmentation of the feedback from group III/IV fibers causes hyper-responsive sympathetic nerve activity (SNA), leading to heart rate (HR) acceleration and hyperventilation [7,8,9].

Signals that ultimately trigger stimulation of venous dilation enter the cardiovascular center of the medulla oblongata through two pathways. One is through group III that stimulates the mechanoreceptor of connective tissue near the veins, and the other is through a fiber of group IV that stimulates the metaboreceptor of the venules and small lymphatic vessels [10,11,12]. Edema associated with venous dilation is involved in the exaggeration of afferent information. A previous study has shown that a venous occlusion technique can produce dilated veins, and cause an increase in ventilation at rest and during exercise [13]. Therefore, it is reasonable to assume that edema is associated with an exaggeration of feedback information from group III/IV fibers. 

Overactivity of SNA may play an important role in developing hypertension and related cardiovascular diseases [14]. Furthermore, chronic venous insufficiency (CVI), a common cause of edema, is associated with an increased risk of developing cardiovascular disease [15]. However, the underlying mechanisms that contribute to this association are not fully understood. If the afferent feedback associated with venous dilation and edema exaggerates the SNA response, this afferent information mechanism could contribute to a decrease in cardiovascular event risk. However, since edema is caused by multiple factors [16,17], which limits the examination of the effect of venous dilation on the ANS in this clinical population, the venous occlusion technique must be used to study the association between venous dilation and the ANS among healthy participants. To date, venous congestion caused by venous occlusion has not been quantitatively evaluated [9]. As venous blood contains more deoxygenated hemoglobin (HHb) than arterial blood, near-infrared spectroscopy (NIRS) can be used as a quantitative assessment of HHb during the venous occlusion technique. 

Consequently, our aim in this study was to examine the effect of different subsystolic venous occlusion pressures on the ANS response. We applied thigh pressure in healthy young men to induce venous dilations of the lower extremities and used heart rate variability (HRV) as an index of the response. We hypothesized that venous occlusion would cause an increase in SNA-induced HRV and a widening of the area under the curve of HHb (HHb-AUC). The findings supporting this hypothesis could provide insight into the mechanisms that contribute to edema in the ANS.

## 2. Materials and Methods

### 2.1. Participants

The study group included 13 healthy men. The inclusion criteria for this study were as follows: (1) a minimum age of 20 years and (2) self-reported status as a non-smoker. The exclusion criteria for this study included any participants who had a diagnosis of a mental illness, neurological disorder, hypertension, or any other health condition affecting HR. Their mean (±standard deviation) age was 20.4 ± 0.5 years, height 172.2 ± 5.1 cm, weight 65.6 ± 6.2 kg, and body mass index (BMI) 22.1 ± 2.0 kg/m^2^. Participants were asked to avoid caffeine and intense exercise for 24 h before the test session and not to eat for 2 h before the session. 

### 2.2. Experimental Protocol

**Figure 1:**
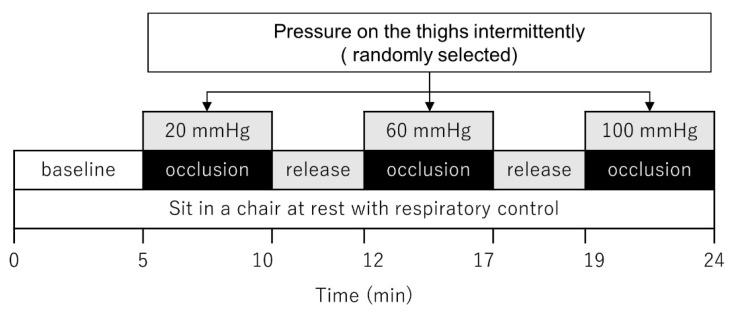
Schematic of the experimental protocol.

The participants were fitted with a 9 cm compression tourniquet for the center of both thighs, which was connected to a rapid cuff inflation device (DTS-2800, Muranaka Medical Instruments, Osaka, Japan). Before the testing session, the cuffs were inflated to ensure the absence of pain or other discomfort. The experimental protocol is presented in Figure 1. Participants maintained a seated resting position for 5 min before application of the first occlusion pressure. The cuff was inflated and the compressive pressure was maintained for 5 min at each of the three preset occlusion pressures, 20, 60, and 100 mmHg below the systolic pressure, respectively. The three occlusion pressures were randomly varied among participants, with a 2 min rest period between the different levels of applied pressure. A respiratory rate of 0.25 Hz (2 s exhalation and 2 s inhalation) was maintained throughout the rest and compression periods to reduce errors caused by the influence of HRV due to respiratory sinus arrhythmia [18]. The respiratory rate was controlled using an electronic metronome set to 60 bpm to provide auditory feedback. 

### 2.3. Physiological Data Collection

**Figure 2:**
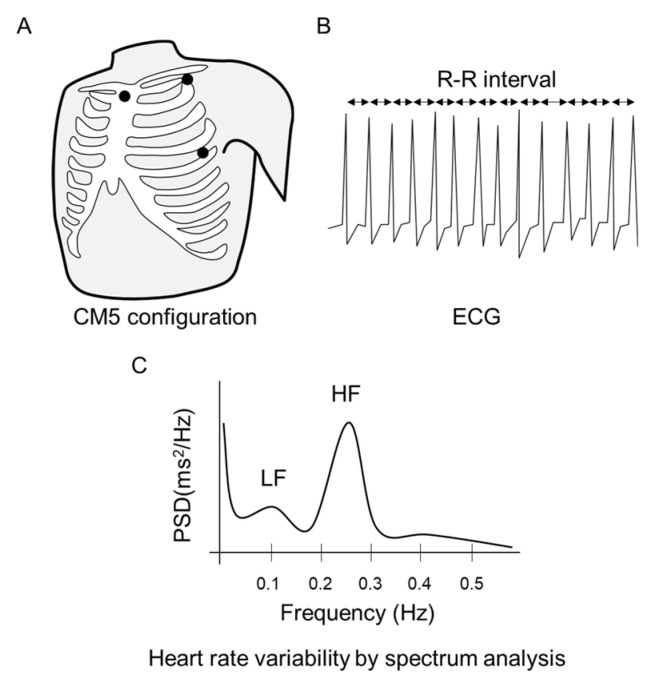
Overview of the electrocardiogram and heart rate variability. ECG, electrocardiography; LF, low frequency; HF, high frequency.

Electrocardiography (ECG) was performed using one lead in a standard CM5 configuration with three silver chloride monitoring electrodes placed on the chest (Figure 2A). ECG traces were recorded using a polygraph system (RMT-1000MG; Nihon Kohden, Tokyo, Japan) and Labchart Pro 8 (ADInstruments, Dunedin, New Zealand), at a sampling frequency of 1 kHz. Spectral analysis was performed separately for each 5 min epoch of the R-R interval sequence, using a fast Fourier transform algorithm (Figure 2B,C). The frequency domain analysis performed in this study was designed to evaluate various components of the electrocardiogram signal, including total power (TP), very low frequency (VLF), low frequency (LF: 0.04–0.15 Hz), and high frequency (HF: 0.15–0.40 Hz) components. The LF and HF components were subsequently normalized using the following equations: LFnu = LF/(TP − VLF) and HFnu = HF/(TP − VLF). The LF/HF ratio was calculated as the LF power/HF power, providing an indirect estimate of the balance between the sympathetic and parasympathetic branches of the ANS, with higher values implying greater sympathetic or less vagal influence [19]. 

**Figure 3:**
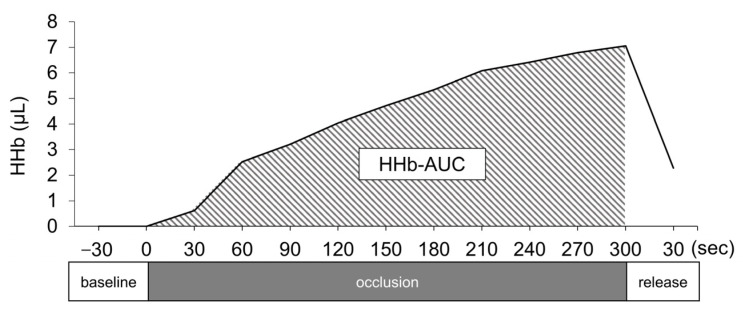
Overview of analysis of the area under the curve of deoxygenated hemoglobin. HHB, deoxygenated hemoglobin; HHb-AUC, area under the curve of HHb.

Systolic (SBP) and diastolic (DBP) blood pressure and mean arterial pressure (MAP) were measured noninvasively from the right upper arm using an automated sphygmomanometer (Tango M2, SunTech Medical, Morrisville, NC, USA), with values obtained at 1 min intervals. NIRS (Portamon, Artinis Medical Systems, Elst, The Netherlands), with different wavelengths, was used to measure HHb. The NIRS probe and detector were placed longitudinally over the maximum volume of the right triceps surae (calf) muscle. An opaque wrapping was used to fix the NIRS sensors in place and eliminate the effects of direct light on measured values. The NIRS signal was sampled at 1 Hz using the Oxysoft system (Artinis Medical Systems, Elst, The Netherlands). To calculate the cumulative increase in HHb in the calf with venous occlusion, the mean value of HHb was calculated for 30 s before applying compressive force, and the HHb-AUC was calculated over a 5 min period after venous occlusion (Figure 3). Although the HHb count is a relative value [20], converting the HHb-AUC provides an absolute comparison between conditions.

### 2.4. Statistical Analysis

Data are presented as the mean ± standard error. Differences in measured outcomes (SBP, DBP, MAP, HR, LF, HF, and LF/HF) were evaluated using repeated measure linear mixed models with occlusive pressures as fixed factors. HHb-AUC was evaluated using a mixed linear model with the condition as a fixed factor. For the analyses, the participants were entered as random effects. The post hoc tests were multiple comparisons using the sequential Bonferroni correction to adjust for multiple comparisons, adjusted for BMI and age.

All statistical analyses were performed with the SPSS version 26 statistical software package for Windows (IBM Corp, Armonk, NY, USA). 

## 3. Results

None of the participants reported pain during venous occlusion. The measured cardiovascular variables and HRV parameters during all phases of the experiment are reported in Table 1. 

**Figure 4:**
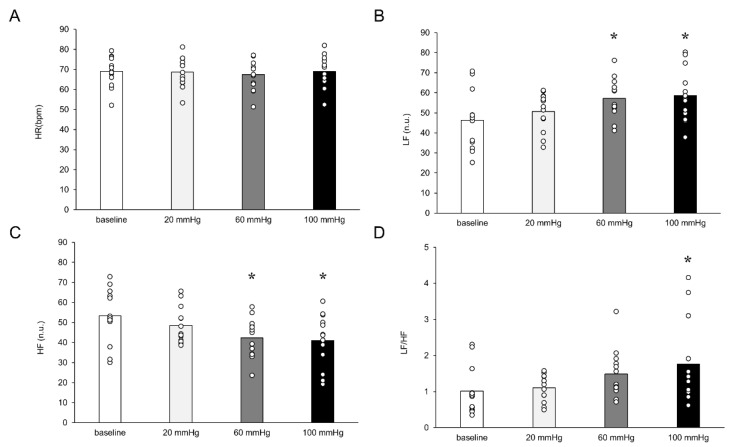
Effects of venous occlusion on heart rate variability. Shown are the (**A**) heart rate (HR), (**B**) low-frequency (LF), (**C**) high-frequency (HF) components of heart rate variability (HRV), and (**D**) the resultant effect on the LF/HF balance. White dots indicate the data for each participant. LF increased and HF decreased at occlusion pressures of 60 and 100 mmHg (*p* < 0.05). The LF/HF balance increased at an occlusion pressure of 100 mmHg (*p* < 0.05). * *p* < 0.05 compared to resting baseline.

**Figure 5:**
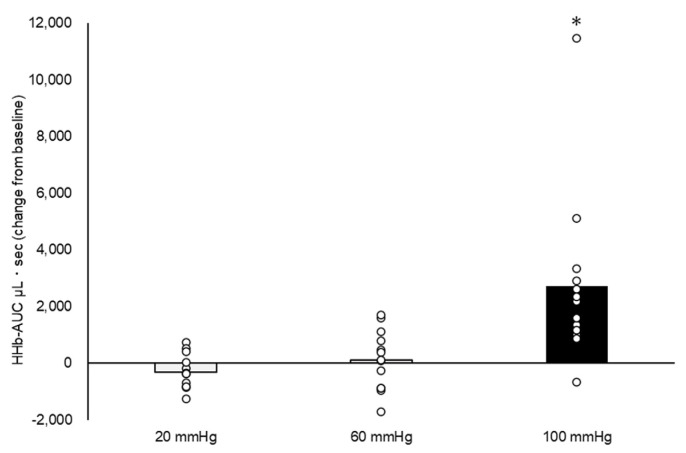
Area under the curve of deoxygenated hemoglobin (HHb-AUC) for the three occlusion pressures. White dots indicate the data for each participant. There was a significant increase in the HHb-AUC for the 100 mmHg pressure compared with the 20 mmHg and 60 mmHg (*) pressures (*p* < 0.05). Between-group differences were evaluated using a Bonferroni post hoc test.

**Figure 6:**
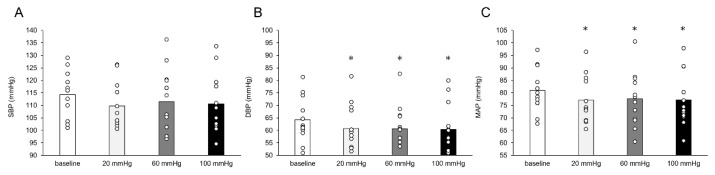
Effects of venous occlusion on blood pressure changes shown are the (**A**) systolic blood pressure (SBP), (**B**) diastolic blood pressure, and (**C**) mean arterial pressure (MAP). White dots indicate the data for each participant. DBP and MAP decreased at all occlusion pressures from baseline. * *p* < 0.05 compared to resting baseline.

There was no difference in HR from baseline to occlusion (all *p* < 0.05). There was a significant mean change in increased LF and decreased HF at occlusion pressures of 60 mmHg (LF, 10.9 ± 3.5 nu; HF, −10.9 ± 3.4 nu) and 100 mmHg (LF, 12.4 ± 3.5 nu; HF, −12.2 ± 3.4 nu), all *p* < 0.05 (Figure 4). At an occlusive pressure of 100 mmHg, changes in HRV induced a significant change in the LF/HF balance (mean difference from rest, 0.74 ± 0.25, *p* < 0.05). The resulting effects of venous occlusion on HHb-AUC are shown in Figure 5. Although the mean difference in HHb-AUC was not significant between the 20 and 60 mmHg occlusive pressures (422.8 ± 770.2 μL s; *p* > 0.05), the difference was significant between 20 and 100 mmHg (3018.5 ± 770.2 μL s) and between 60 and 100 mmHg (2595.7 ± 770.2 μL s), both *p* < 0.05. MAP decreased significantly at the three levels of venous occlusion pressures compared to baseline resting, with a mean decrease of 3.8 ± 1.1 mmHg at an occlusion pressure of 20 mmHg, 3.9 ± 1.1 mmHg at 60 mmHg, and 3.4 ± 1.2 mmHg at 100 mmHg (Figure 6C, all *p* < 0.05). This decrease in MAP appeared to be driven by a change in DBP, with a mean difference in DBP from baseline of 3.8 ± 1.2 mmHg at an occlusion pressure of 20 mmHg, 3.6 ± 1.2 mmHg at 60 mmHg, and 3.7 ± 1.2 mmHg at 100 mmHg (Figure 6B). 

## 4. Discussion

As a novel contribution, our findings identified an increase in the LF/HF ratio under a compressive pressure of 100 mmHg, with a resulting increase in HHb-AUC. These findings supported our hypothesis that increased afferent feedback through group III/IV sensory fibers of the lower limb veins, induced by the venous occlusion technique, increases the cardiac response of SNA.

The feedback to the circulatory system through group III/IV fibers helps maintain blood perfusion to exercising skeletal muscles by modulating cardiac output and vascular resistance during exercise [1,21]. In our study, the HRV balance shifted to SNA dominance with a venous occlusion pressure of 100 mmHg, with an increase in LF and a decrease in HF with a venous occlusion ≥60 mmHg. Previous studies have shown that the LF component is associated with a response of the baroreflex system, with an increase in the LF component when standing compared to lying supine [22,23,24]. Cardiovascular control via baroreflex instantly increases blood pressure to maintain systemic perfusion [25,26,27]. Therefore, our results suggested that baroreflex may be elicited by 60 and 100 mmHg pressurization, due to the reduced venous return caused by venous occlusion. Furthermore, venous occlusion ≥60 mmHg led to a decrease in the HF component. 

Stimulation of group III/IV afferent fibers by dilation of leg veins induced by thigh compression has previously been reported [10,12]. In particular, group III afferents, which sense mechanical stimuli from dilated veins, increase HR through a downregulation of the vagal response [28,29]. Therefore, feedback from venous dilation of the lower limbs causes a decrease in HF, principally through inputs from group III afferents. However, the present findings indicated that the HR did not exhibit a significant increase. This lack of response may be attributed to the fact that the magnitude of the HR response is contingent upon the extent of group III fiber recruitment. Previous research has established that the recruitment of group III fibers plays a crucial role in modulating cardiovascular responses [30]. In the present study, the recruitment of group III fibers in the lower leg was employed as the intervention. However, it should be noted that in other studies, recruitment of group III fibers has been achieved through the engagement of the entire lower limb.

To our knowledge, our study is the first to evaluate venous dilation using a venous occlusion technique and to quantify the resulting effect on HHb-AUC (Figure 5). HHb-AUC should be considered an outcome measure in future studies, as it can be evaluated non-invasively using NIRS and provides a proxy measure of microvascular flow, and thus of tissue hemodynamics of tissue microvascular flow [31,32]. In a previous study, there was a decrease in the HHb level of the leg during walking compared to the resting state in healthy participants, but with an opposite response in patients with CVI [33]. The elevation in HHb levels observed in the legs of patients with CVI during walking may be attributed to the combination of increased venous pressure and stasis resulting from malfunctioning venous valves and decreased muscle contractility in the surrounding veins, which leads to venous dilation [34,35]. 

These findings regarding the correlation between CVI and HHb provide support for our utilization of HHb-AUC as a method to evaluate the effects of venous dilation. Our use of the AUC, rather than the HHb level, allows for absolute comparisons between conditions. Our results showed that a 100 mmHg cuff occlusion increased the HHb-AUC compared with a 20 mmHg and 60 mmHg cuff occlusion. The venous pressure in the normal upright position at rest is 80 mmHg in the posterior tibial vein [36]; therefore, the applied pressure of 100 mmHg is higher than the venous pressure in the lower extremities, considered enough to occlude the femoral veins and induce venous dilation. The assessment of HHb-AUC at 100 mmHg cuff pressure provides a robust method to evaluate the contribution of group III/IV sensory fiber feedback on the cardiac response. Furthermore, the 20 mmHg pressurization resulted in decreased HHb-AUC. Agu et al. [37] reported that wearing compression stockings (mean ankle pressure of 16.8 to 29.1 mmHg) decreases venous pooling during walking for 5 min. Furthermore, HHb falls during walking due to muscle pumping [33]. Therefore, 20 mmHg pressure increased venous return, resulting in a HHb-AUC of decreased value.

We noted a decrease in DBP and MAP for all occlusion pressures compared to the resting baseline (Figure 6B,C). This decrease in DBP reflects a decrease in diastolic blood flow due to mechanical deformation of the artery caused by femoral compression [38], which can lead to the production of metabolites that stimulate group III/IV fibers. However, a previous study showed a higher arterial flow in the lower leg at 80–90 mmHg compared with 50–70 mmHg of pressure in the thigh in healthy participants [39]. Therefore, our study shows that the increase in arterial inflow in femoral compression below arterial pressure counteracts the reduced arterial diameters in the femoral artery. In this sense, the measurements of HRV during the venous dilation by femoral compression used in this study likely reflect an inhibition of the metaboreflex by hypoxia-induced metabolites. Similar reductions in DBP and MAP were observed in all thigh pressures. LF increased with a pressure of 60 and 100 mmHg and did not change with a pressure of 20 mmHg in this study, suggesting that pressure over 60 mmHg may affect baroreflex blood pressure regulation, which requires a detailed evaluation of blood pressure dynamics.

In recent years, a correlation between CVI and cardiac risk factors has been established through the analysis of large cohort studies [15]. However, the underlying mechanisms that link these two conditions remain poorly understood. Our research suggested that edema, resulting from impaired venous function in CVI, may contribute to cardiovascular risk through the involvement of the ANS. This proposed link between autonomic response to increased afferent information, caused by edema, offers a new perspective on the prevention of cardiac disease. 

The strengths and limitations of our study should be considered in the interpretation of the findings. As a strength, HRV changes due to venous dilation were evaluated using a robust methodology. However, it should be acknowledged that there were certain limitations in the study. First, the sample size in this study was relatively small, which may limit the generalizability of our results. However, a linear mixed model was used to account for inter-individual variation by including random effects for participants. Second, the contribution of group III/IV afferent fibers to the cardiac response was inferred, as direct measurement of afference in group III/IV fibers is not feasible in participants. Additionally, we were unable to fully evaluate the ventilatory response to stimulation of group III fibers. This was due to the fact that the study only captured a limited portion of the physiological response to the stimulation of group III fibers, which simulates edema. This limitation was necessary in order to control respiration and allow for a more thorough examination of the cardiovascular effects. Third, the use of a venous cuff occlusion may have led to venous distension and activation of mechanosensitive afferents in skeletal muscle, which could not be fully explained as muscle metabolites were not monitored. Furthermore, it is possible that the metabolic reflex may have been activated, particularly at high cuff pressures where blood perfusion to the muscles may have been compromised. Fourth, the effect of the pressure of the occlusion cuff on changes in intravascular, interstitial, or intramuscular pressure, secondary to the induced venous occlusion, could not be differentiated.

## 5. Conclusions

Our findings indicated that venous dilation, induced by the venous occlusion technique at a pressure of 100 mmHg, exacerbated the sympathetic HRV response. Therefore, induced lower limb edema can affect cardiovascular regulation in healthy male adults through feedback from group III/IV sensory fibers. These findings suggest that edema may elicit a shift towards sympathetic dominance in the autonomic balance. Thus far, there is no literature to support a correlation between edema and changes in HRV. Therefore, further investigation into patients with CVI is needed to obtain clinically meaningful findings.

## Figures and Tables

**Table 1:** Changes in measured heart rate variability and blood pressure variables for the three venous occlusion pressures.

Variable	Baseline	20 mmHg	60 mmHg	100 mmHg	*F* Value	*p* Value
HR, beats/min	69.0 ± 2.2	68.6 ± 2.2	67.4 ± 2.2	68.9 ± 2.2	1.58	0.21
LF, ms^2^	912.0 ± 455.5	910.6 ± 455.5	2237.3 ± 455.5	1732.1 ± 455.5	3.04	0.04
LF, normalized unit	46.3 ± 3.3	50.7 ± 3.3	57.2 ± 3.3 *	58.7 ± 3.3 *	5.35	0.00
HF, ms^2^	1143.7 ± 289.0	923.7 ± 289.0	1555.0 ± 289.0	1123.2 ± 289.0	1.47	0.24
HF, normalized unit	53.2 ± 3.2	48.6 ± 3.2	42.4 ± 3.2 *	41.1 ± 3.2 *	5.55	0.00
LF/HF	1.1 ± 0.2	1.1 ± 0.2	1.5 ± 0.2	1.8 ± 0.2 *	3.88	0.02
SBP, mmHg	114.3 ± 3.0	109.7 ± 3.0	111.5 ± 3.0	110.6 ± 3.0	2.84	0.05
DBP, mmHg	64.3 ± 2.6	60.7 ± 2.6 *	60.6 ± 2.6 *	60.5 ± 2.6 *	4.70	0.01
MAP, mmHg	81.0 ± 2.7	77.1 ± 2.7 *	77.5 ± 2.7 *	77.2 ± 2.7 *	5.35	0.00

Values reported as the mean ± standard error. HR, heart rate; LF, low-frequency component; HF, high-frequency component; nu, normalized unit; SBP, systolic blood pressure; DBP, diastolic blood pressure; MAP, mean arterial pressure. * *p* < 0.05, baseline vs. cuff occlusion.

## Data Availability

Not applicable.
